# Prevalence, Severity and Treatment Needs of Molar Incisor Hypomineralization Among a Cohort of Lebanese Children: A Cross-Sectional Study

**DOI:** 10.3390/children13050708

**Published:** 2026-05-21

**Authors:** Ayah Khodor, Ahmad Tarabaih, Mohammad Alkilzy

**Affiliations:** 1 Division of Pediatric Dentistry, Faculty of Dentistry, Beirut Arab University, Beirut P.O. Box 11-5020, Lebanon; ayahkhodor@hotmail.com (A.K.); a.tarabaih@bau.edu.lb (A.T.); 2 Department of Pediatric Dentistry, University of Greifswald, 17489 Greifswald, Germany

**Keywords:** molar incisor hypomineralization, MIH, prevalence, severity, treatment needs, children

## Abstract

**Highlights:**

**What are the main findings?**

- Considerable MIH prevalence of 17.14% among study cohort.
- Although males exhibited a greater number of affected teeth and increased severity, these differences were not statistically significant.

**What are the implications of the main findings?**

- Prevalence rate highlights MIH as significant oral health concern that warrants systematic public monitoring.
- Early identification is essential to apply preventive strategies and early treatment approaches to avoid MIH cases from progressing into severe conditions that require invasive procedures.

**Abstract:**

Objectives: The aim of the study was to assess the prevalence, severity and treatment needs of molar incisor hypomineralization (MIH) among a cohort of children in Lebanon. Methods: A cross-sectional study was performed in Akkar district in northern Lebanon on 1237 school children between 10 and 12 years old (mean age 11.11 ± 0.80 years) who were recruited from eight private schools. Clinical evaluations were performed after teeth cleaning and drying using sterile gauze. Examiners assessed all teeth present in the oral cavity using the MIH index of the EAPD. If MIH was diagnosed, a further grading was made according to the MIH-TNI index. Results: In total, 212 children (17.14% [95% CI:15.0, 19.2]) were diagnosed with MIH. Of these, 200 children with completed documentation were included in the further statistical analysis. Out of the affected cases, mild lesions were the most common at 41% (n = 82). Although males exhibited higher cumulative numbers of affected teeth (52.5%, n = 267), with notably greater proportions in moderate (17.7%) and severe (16.7%) cases, the difference between genders was not statistically significant (*p* = 0.069). Treatment needs increased with severity, in which mild lesions primarily required preventive care (41.0%), while moderate lesions required restorative treatments (34.0%); in contrast, severe lesions (25.0%) often necessitated extensive interventions, such as crowns, pulp therapies or even extractions. Although descriptive patterns suggested an increase in more complex treatment needs in older age groups, the association was not statistically significant (*p* = 0.26). Conclusions: The prevalence of MIH observed in this cohort study aligns with internationally reported values. Early detection, preventive management, and timely restorative interventions are essential to minimize the long-term impact of MIH.

## 1. Introduction

Developmental Defects of Enamel (DDEs) represent disturbances in the formation and mineralization of hard dental tissues that occur during odontogenesis, from approximately the 16th week of gestation until the age of five years. These non-carious defects may affect both primary and permanent dentitions, thereby negatively influencing oral health and quality of life [1,2]. Among these defects, molar incisor hypomineralization (MIH) is a well-recognized qualitative enamel anomaly involving one to four first permanent molars, often associated with permanent incisors [3]. It is characterized by demarcated opacities ranging in color from white and cream to yellow or dark brown, with or without post-eruptive enamel breakdown (PEB). The hypomineralized enamel exhibits increased protein and carbonate content, along with reduced mineral density, hardness, and elastic modulus [4]. Clinically, MIH-affected teeth often present with hypersensitivity, rapid caries progression, and difficulty in achieving adequate local anesthesia [5].

The global prevalence of MIH has been estimated at an average of 15.50%, with reported values ranging from 0.6% and 46.6% [6]. In the Middle East, prevalence rates are comparable, varying between 2.3% and 40.7%, with a regional average of 15.05% [7]. In Beirut, Lebanon, the prevalence of MIH was reported to be 26.7% in 2020 [8].

The clinical presentation of MIH is often asymmetrical, which has led to hypotheses of a systemic disruption during amelogenesis, but the condition is now understood as multifactorial with both genetic and environmental contributors [9]. The multifactorial aetiology of MIH is globally acknowledged, likely driven by gene–environment interactions [10]. This multifactorial condition may result from systemic factors such as acute or chronic diseases or exposure to environmental contaminants during the last trimester of pregnancy and early childhood. The number of affected teeth appears to correlate with the timing of systemic disorders, with children experiencing prenatal, perinatal, and postnatal complications exhibiting progressively greater numbers of affected teeth, respectively [9,10].

Prenatal determinants include maternal sickness, while perinatal factors encompass premature birth and low birth weight. Postnatal factors include early childhood diseases, such as febrile conditions, infectious diseases, and antibiotic use [11].

Histological investigations indicate that MIH-affected enamel has a significantly reduced mineral density, approximately 20–22% lower than that of sound enamel [12]. Additionally, the incorporation of carbonate ions increases enamel solubility. Structurally, the hydroxyapatite crystal arrangement appears disorganized, with morphological alterations leading to increased porosity. Mechanically, this results in reduced hardness and elastic modulus, rendering the enamel more fragile [13].

Diagnostic criteria for MIH were established in Athens in 2003 by the European Academy of Pediatric Dentistry (EAPD) [14]. These include demarcated opacities, hypersensitivity, post-eruptive enamel breakdown, atypical restorations, tooth loss due to MIH-related complications, and unerupted first permanent molars associated with underlying pathology [15].

Management of MIH in children is essential, as its clinical manifestations and complications can vary considerably. Early diagnosis is crucial to enable appropriate care and the implantation of recall programs, thereby improving the likelihood of achieving both aesthetic and functional rehabilitation [16].

Given the strong association between MIH and dental hypersensitivity, careful evaluation is required. The MIH Treatment Need Index (MIH-TNI) was therefore developed in 2017 to guide treatment planning based on clinical presentation. This index is based on two key clinical parameters: hypersensitivity and the extent of enamel defects [17].

Based on the MIH-TNI, a structured treatment approach known as the “Wurzburg concept” was developed in the form of a flowchart to assist clinicians in daily practice. This approach includes strategies such as enamel remineralization, prophylaxis, fissure sealing, direct restorative treatments, and long-term management planning. Two parallel flowcharts were designed for patients with low and high caries risk, emphasizing the importance of individualized treatment planning. This concept was updated in 2022 to incorporate non-invasive approaches and temporary modalities [18].

To date, evidence from the Middle East and North Africa (MENA) region remains limited. Clinical observations by pediatric dentists in Lebanon in recent years suggest an apparent increase in the prevalence of MIH. However, comprehensive epidemiological data on the Lebanese pediatric population are lacking, and the associated treatment needs have not been systematically evaluated or formally documented.

Therefore, the present study aimed to assess the prevalence, severity, and treatment needs of MIH in a cohort of Lebanese children.

## 2. Materials and Methods

This cross-sectional study was approved by the Research Ethics Committee of Beirut Arab University (IRB code: 2025-H-0178-D-M-0773). Written informed consent was obtained from parents or guardians prior to participation, following a clear explanation of the study’s aims, risks, and benefits. A total of 1237 children aged 10–12 years were randomly selected from eight private schools in the Akkar District using a stratified cluster sampling method, with equal representation from urban (four schools) and rural (four schools) areas. All invited parents and children agreed to participate in the study with response rate of 100%.

### 2.1. Study Sampling and Sample Size Calculation

The total population of Akkar Governorate is approximately 423,600 people, according to the Central Administration of Statistics (CAS). Based on the national age structure (approximately 9.4% for ages 10–14), the number of children aged 10–12 years in Akkar was estimated at 23,891. The required sample size was calculated using the Raosoft online calculator (https://raosoftcalculator.com/, accessed on 21 November 2024), assuming a 5% margin of error and a 95% confidence interval and yielding a minimum sample of 378 children. In the present study, 1237 children aged 10–12 years were examined.

### 2.2. Patient Selection

Inclusion and exclusion criteria applied for patient selection in this study are summarized in Table 1.

### 2.3. Methodology

All clinical examinations were conducted using a standard dental diagnostic kit, including a mirror No. 5 and a Community Periodontal Index (CPI) probe, as recommended by the World Health Organization. Teeth were cleaned and dried with sterile gauze prior to examination. All teeth present were assessed, and MIH was diagnosed using the short-form MIH index in accordance with the European Academy of Pediatric Dentistry criteria [14]. Care was taken to differentiate MIH from fluorosis and dental caries to minimize misdiagnosis. When MIH was identified (“MIH Yes’’), lesions were further graded on a scale from 1 to 4 [16].

The MIH-TNI was used for both diagnostic and treatment planning purposes, as well as for epidemiological assessment [18]. The dentition was divided into six sextants to allow systematic evaluation, facilitate identification of lesion distribution (localized versus generalized), and assess regional severity. Posterior teeth typically present with greater treatment needs than anterior teeth.

Assessments were conducted in a clockwise direction, beginning with the maxillary right quadrant. The highest score recorded within each sextant was used to determine severity. Overall, MIH severity was defined by the highest score observed across all sextants [14].

Measurements taken in every sextant led to a yes or no decision:

- “No-decision”: Status/Index O: no signs of MIH are found, clinically healthy.
- “Yes-decision”: Status/Index 1 or higher: signs of MIH were found.

Following confirmation of MIH, treatment needs were determined using the Wurzburg MIH Concept (Version 2.0), which expands upon the original 2016 framework. This updated version includes non-invasive strategies, temporary treatment options, and specific approaches for incisor management, covering interventions from preventive care to extraction [18]. The index has 5 groups: index 0: no MIH; index 1: MIH without breakdown and without hypersensitivity; index 2: MIH with breakdown and without hypersensitivity divided into: 2a: extension of defect less than 1/3; 2b: extension between 1/3 to 2/;2c: extension more than 2/3 or/and defect close to the pulp or extraction or atypical restoration; index 3: MIH without breakdown but with hypersensitivity; and index 4: MIH with breakdown and with hypersensitivity divided to 4a, 4b, and 4c, as described in index 2.

The treatment plan modalities based on MIH-TNI classifications are divided to 6 therapy groups: therapy A: prophylaxis/regeneration (treatment codes: A1: at home fluoride; A2: in office fluoride varnish); therapy B: non-invasive therapy (treatment codes B1: fissure sealant; B2: glass ionomer cement of low viscosity; B3: bleaching; B4: microabrasion; B5: infiltration; B6: etch-bleach-seal); therapy C: short-term temporary therapy (treatment codes: C1: GIC; C2: GIC plus othroband; C3: SDF plus GIC; C4: SDF); therapy D: long-term temporary therapy (treatment code D: crowns); therapy E: permanent therapy (treatment codes E1: direct restoration; E2: indirect restoration); and therapy F: extraction (treatment code F) [18].

### 2.4. Examiner Reliability

The examiner underwent training and calibration in the Division of Pediatric Dentistry at Beirut Arab University. Calibration was performed on 20 children who were excluded from the main study. All assessments were conducted twice by the same examiner, with a 20 min interval between examinations. The results were compared blindly to assess intra-examiner reliability. Cohen’s kappa coefficient was calculated at 0.85, indicating strong agreement.

### 2.5. Statistical Analysis

Following data collection, all records were de-identified and coded to ensure participant anonymity using Microsoft Excel 2016. Statistical analysis was performed using the Statistical Package for the Social Sciences (SPSS), version 30.0. Descriptive statistics were used to summarize frequencies and percentages. The prevalence of MIH was calculated with a 95% confidence interval (CI). The association between categorical variables was assessed using the chi-square test with statistical significance set at *p* < 0.05.

## 3. Results

### 3.1. Sample Description and Prevalence of MIH

Data collection was conducted over a six-month period: from October 2024 to April 2025.

**Figure 1:**
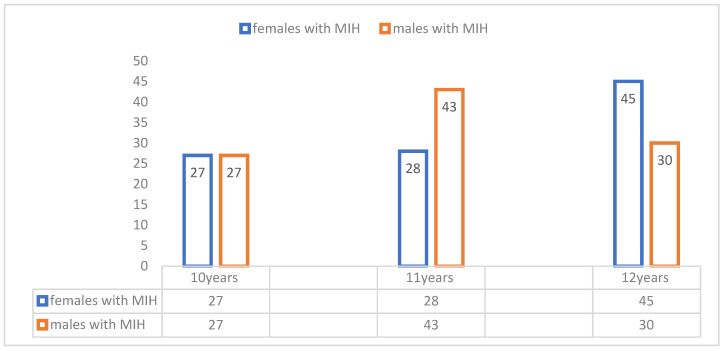
Distribution of children with MIH according to gender and age.

In total, 1237 children aged between 10 and 12 years (mean age 11.11 ± 0.80 years) were examined in eight schools. The prevalence of MIH in the study population was 17.14% (212/1237), with a 95% confidence interval of 15.0% to 19.2%. Complete diagnostic information was available for 200 children, who comprised the study population for subsequent analysis. The age groups were divided into three age groups of 10, 11 and 12 years old (Figure 1).

The distribution of children with MIH varied across age and gender. Ten-year-old males and females were equally affected by MIH (13.5% each, n = 27 each). In the 11-year-old group, MIH prevalence was higher in males (21.5%, n = 43) than in females (14%, n = 28). This pattern shifted in 12 year olds, with 22.5% (n = 45) females compared to 15% (n = 30) males.

### 3.2. Severity of MIH

The distribution of MIH severity across age shows differences in how the condition presents among 10, 11, and 12 year olds. Mild MIH showed a higher prevalence in the higher age groups of 11 and 12 year olds. In contrast, moderate MIH was almost equal in all age groups of 10, 11, and 12 year olds (11.0%, 11.5%, and 11.5%, respectively) out of 68 total cases. In contrast, severe MIH and more complex cases were seen in the 12-year-old group [Table 2]. The distribution was analyzed using the chi-square test. Although descriptive differences were observed, the association between age and MIH severity was not statistically significant (*p* = 0.72).

The distribution of MIH severity by gender revealed notable differences in how the condition presented among boys and girls. Girls showed a higher prevalence of mild cases at 60% (n = 49). Whereas moderate and severe MIH cases were relatively more common among boys, comprising 56% (n = 38) and 58% (n = 29) of cases, respectively [Table 3]. The distribution was analyzed using the chi-square test. Although differences were observed in the distribution of severity between boys and girls, the association was not statistically significant (*p* = 0.069).

### 3.3. Total Number of Teeth Affected

**Figure 2:**
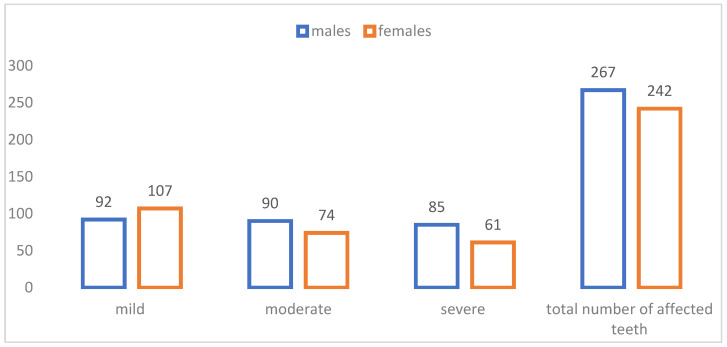
Distribution of the total number of MIH teeth according to severity and gender.

In the study sample, a total of 509 teeth were affected with MIH, distributed in distinct patterns between females and males. Mild cases were the most frequently observed, with females accounting for 21% (n = 107). Moderate and severe cases were higher in males (18%, n = 90) and (17%, n = 85), respectively. However, when assessing the total number of affected teeth, males exhibited a relatively higher total number of affected teeth, with nearly 52.5% of all cases compared to 47.5% in females (Figure 2).

### 3.4. Distribution of MIH Treatment Needs According to Gender and Age

In the study sample, the MIH Treatment Needs Index, MIH-TNI, varied between girls and boys. In females, most cases (50.4%) (n = 62) showed MIH-TNI 1, followed by MIH-TNI 2b at 14.6% (n = 18) and MIH-TNI 2a at 13.0% (n = 16). Severe treatments such as MIH-TNI 4b and MIH-TNI 4c were relatively less frequent, accounting for 3.25% (n = 4) each. In males, MIH-TNI 1 was the most prevalent category (38.0%, n = 51), while MIH-TNI 2a showed a higher relative proportion compared to females (20.1%, n = 27). Severe treatment needs were more common in males, particularly MIH-TNI 4c (10.4%, n = 14) and MIH-TNI 4b (5.22%, n = 7) [Table 4]. Although descriptive patterns suggested an increase in more complex treatment needs in older age groups, the association was not statistically significant (*p* = 0.26).

A total of 257 sextants were assessed, of which 123 belonged to females, while 134 belonged to males. Both males and females demonstrated an increasing trend in MIH-TNI 1 with age, with the highest counts at 12 years (females, n = 30; males, n = 12). When combining both genders, MIH-TNI 1 represented the majority of cases (43.9%, n = 113), followed by MIH-TNI 2a (16.7%, n = 43) and MIH-TNI 2b (13.6%, n = 35). More severe cases, such as MIH-TNI 4c and MIH-TNI4b, accounted only for 6.2% (n = 16) and 4.6% (n = 12) of the total cases, respectively.

### 3.5. Distribution of MIH Treatments by Tooth

The total number of affected teeth was 509. According to the recommended therapy plan based on the MIH-TNI in patients with high caries risk, the most common treatment modality overall was B1, with 206 cases (40.4%), followed by E1 and D with 132 cases (26%) and 97 cases (19%), respectively. In contrast, other modalities, such as B5, B2, and F (8.2% (n = 42), 2.4% (n = 12), and 1.6% (n = 8), respectively), were less frequent, while C1 and C3 were rarely indicated (0.6% and 0.8%, respectively). This indicates that predominant treatments were preventive and restorative interventions, such as fissure sealants (B1), stainless-steel crowns (D), and restorations (E1). However, more complex approaches, such as indirect restorations (E2) and extractions (F), were reserved for fewer cases.

Among males, B1 was the most common treatment applied (20.4%, n = 104), followed by E1 (14%, n = 71) and D (10.8%, n = 55). In females, B1 was also the most frequent (20%, n = 102), followed by E1 (12%, n = 61) and B5 (4.5%, n = 23). More invasive modalities such as D, E2, and F were less frequently applied, while C1 was not recorded among females [Table 5].

A chi-square test of independence was conducted to assess the association between gender and the distribution of enamel defect types. The test showed no statistically significant association between gender and defect category (*p* = 0.288). This indicates that the prevalence of individual defect types did not differ significantly between males and females in the study sample.

Age-wise, there was a clear trend toward increasingly complex treatment needs with age. In 10 year olds, conservative treatments such as B1 dominated, with limited use of more advanced approaches. By age 11 years, an increase in E1 and D was observed, reflecting a shift toward more invasive restorations. In 12 year olds, females showed notably higher counts of B5 (n = 18) and F (n = 2), while males showed higher use of E1 (n = 48). Additionally, the proportion of advanced treatments, such as D, E1, E2, and F, increased further (26.4% combined in males and 21.2% combined for females). This progression indicates that as age increases, the severity of MIH-related defects tends to require more complex and invasive management.

## 4. Discussion

MIH has become an increasing focus of interest in pediatric dentistry, particularly with regard to its diagnosis and management challenges. To overcome these challenges, the MIH Treatment Need Index (MIH-TNI), incorporated within the Wurzburg concept, was developed to standardize severity-based treatment planning [18]. Despite its clinical effectiveness, epidemiological studies focusing specifically on treatment needs remain limited, with most research primarily addressing prevalence and clinical presentation [19,20,21,22]. This gap restricts meaningful comparisons across populations and highlights the importance of structured indices such as the MIH-TNI in both clinical and research settings [18].

In Lebanon, previous reports focused on MIH prevalence, clinical characteristics, and associated risk factors, with little attention given to treatment needs [23]. Moreover, the inclusion of younger age groups (e.g., 7–9 years) limits understanding of disease progression and evolving treatment needs. Thus, the current study aimed to evaluate MIH prevalence, severity, and treatment needs among a well-distributed sample of preadolescents aged between 10 and 12 years.

Clinical evaluations were performed in accordance with the WHO guidelines, where mirror No. 5 provides optimal visibility, light reflection, and magnification, which are essential for identifying color changes and demarcated opacities associated with MIH. The CPI dental probe allows gentle tactile examination, helping to distinguish between post-eruptive enamel breakdown and intact but discolored enamel without causing iatrogenic damage to the affected surface.

The prevalence of MIH in Akkar–Lebanon (17.14%) was lower than that previously reported in Beirut (26.7%) [23], yet it remained within the regional range of 5% to 40% and slightly above the global estimates (15.5%) [6]. These variations may reflect differences in environmental exposures, socio-economic factors, or diagnostic thresholds.

The literature provides different findings regarding the comparison of MIH severity and, consequently, the complexity of treatment needs between preteen males and females [24]. While many studies, including a global meta-analysis, report no significant difference in the overall prevalence or severity of MIH between genders in preteen populations, suggesting similar overall treatment demands based on defect extent and clinical signs, some regional studies have indicated that females may be more likely to present with severe lesions in the permanent first molars compared to males [25,26,27]. Conversely, other studies have found no significant differences in severity or affected teeth distribution by gender [28,29]. In the presented study, although males exhibited a greater number of affected teeth and increased severity, these differences were not statistically significant (*p* = 0.288). Treatment needs in these age groups are reported in many studies, dictated primarily by the severity and the amount of enamel loss, with severely affected teeth requiring more complex, invasive interventions, such as full coverage restorations using stainless steel or zirconia crowns or even extraction, as opposed to preventive measures such as sealants or fluoride application for mild cases [30,31,32]. These findings are in line with the results of the presented study and were to be predicted according to the nature and the extent of the lesions and the recommended therapies in the MIH-TNI index.

Mild cases were the majority in this study (39.1%). Males exhibited higher cumulative numbers of affected teeth (n = 267) in comparison to females (n = 242), specifically in moderate and severe cases, unlike mild cases, where females were more affected than males. The predominance of mild MIH aligns with several former studies confirming that early-stage lesions are more common in preteens [19,23]. However, some studies showed higher percentages of moderate and severe cases [25,33]. A study conducted in Brazil in 2025 disagreed with our results, in which it revealed that 47.7% of the studied population were of mild and moderate cases, while 52.3% were of severe and very severe cases [34]. In the regional comparisons, the severity forms observed in this study align with reports from the MENA region, where most MIH cases presented as mild defects, mainly demarcated opacities, while fewer cases presented moderate to severe involvement. Studies from countries such as Saudi Arabia and Egypt similarly reported that although mild lesions were more frequent, severe cases characterized by post-eruptive enamel breakdown and hypersensitivity were common and contributed significantly to treatment needs [35,36]. In contrast, another study from neighboring country Jordan showed that the MIH-affected participants had more severe lesions than mild/moderate lesions, 35% (n = 65) had mild/moderate lesions, whereas more than half (65%, n = 121) had severe lesions [37]. Any differences between our findings and regional data might be related to variations in diagnostic criteria and scoring systems, as well as population and environmental factors.

The high prevalence observed in males contrasts with some reports showing either no gender difference [22] or higher occurrence in females [25,26,27]. In the presented study, although males exhibited higher cumulative numbers of affected teeth with 52.5% (n = 267), particularly in moderate and severe cases, which were 17.7% and 16.7%, respectively, no statistically significant association between sex and severity was demonstrated. Treatment needs increased with severity, in which mild lesions primarily required preventive care, while moderate lesions required restorative treatment, whereas severe lesions often necessitated invasive interventions such as crowns, pulpal therapies, or even extractions. This difference may be due to timing variations in enamel formation, as permanent molars in males erupt slightly later than in females, thus extending the period during which ameloblasts are susceptible to systemic interactions [2]. Furthermore, behavioral differences between the genders, such as higher rates of trauma or dietary variation in males, can lead to increased lesion destruction [7].

Regarding the treatment needs, there was a clear increase towards more invasive treatment procedures with age. In 10 year olds, conservative treatments dominated with limited use of the invasive approaches. By age 11, there was a shift toward more extensive restorations. In contrast, at the age of 12 years, the total proportion of advanced treatments, such as crowns, complex restorations, and extractions, increased to 26.4% and 21.2% in males and females, respectively. As the child grows, teeth become more susceptible to post-eruptive breakdown, where hypo-mineralized enamel is more porous and fragile [1,2,3,4]. In addition, exposure to environmental factors, including acidic foods, mechanical stress, and poor oral hygiene, worsens enamel defects over time. Delayed or inadequate treatment of initially mild lesions may also contribute to the progression toward moderate or severe forms, thus increasing treatment complexity [38].

## 5. Conclusions

The presented study provides valuable insight into the prevalence, severity, and treatment needs and modalities of MIH in preteens. Treatment needs are closely linked to lesion severity, as expected from MIH-TNI. Therefore, early identification, preventive care, and timely restorative interventions are critical to reduce the long-term impact of MIH.

## 6. Limitations

The findings of this study should be interpreted with caution when considering their broader applicability. As the sample was restricted to children enrolled in private schools within the Akkar District, the results may not be generalizable to public-school students or to the wider pediatric population in Lebanon. Differences in socioeconomic status, access to healthcare services, and educational environments between private and public-school settings may influence oral health outcomes and related factors.

## Figures and Tables

**Table 1:** Inclusion and exclusion criteria for patient selection.

Inclusion Criteria	Exclusion Criteria
Children with fully erupted first permanent molars and incisors	Presence of crowns or orthodontic bands
Signed informed consent from their parents	Ongoing orthodontic treatment
	Systemic conditions associated with dental malformations, dental fluorosis, amelogenesis imperfecta
	History of dentoalveolar trauma

**Table 2:** Distribution of MIH severity among age groups in numbers and % of children.

Severity	Age			
	10 years	11 years	12 years	Grand Total
Mild	19 (9.5%)	31 (15.5%)	32 (16.0%)	82 (41.0%)
Moderate	22 (11.0%)	23 (11.5%)	23 (11.5%)	68 (34.0%)
Sever	12 (6.0%)	17 (8.5%)	21 (10.5%)	50 (25.0%)
Grand Total	53 (26.5%)	71 (35.5%)	76 (38.0%)	200 (100%)

**Table 3:** Distribution of MIH severity in boys and girls among the studied population.

MIH Severity	Boys n (%)	Girls n (%)
Mild	33 (40%)	49 (60%)
Moderate	38 (56%)	30 (44%)
Severe	29 (58%)	21 (42%)

**Table 4:** MIH treatment needs distribution by age and gender across the affected sextants. MIH-TNI is explained in the Methodology Section.

Females					
MIH-TNI Index n (%)	10 Years	11 Years	12 Years	Total Count	Count Main MIH-TNI
MIH-TNI 1	14 (45.1%)	18 (51.40%)	30 (52.60%)	62 (50.40%)	62 (50.40%)
MIH-TNI 2a	7 (22.6%)	4 (11.40%)	5 (8.80%)	16 (13.00%)	43 (34.90%)
MIH-TNI 2b	4 (12.90%)	7 (20.00%)	7 (12.30%)	18 (14.60%)	
MIH-TNI 2c	2 (6.50%)	2 (5.70%)	5 (8.80%)	9 (7.30%)	
MIH-TNI 3	0 (0.00%)	0 (0.00%)	4(7.00%)	4 (3.25%)	4 (3.25%)
MIH-TNI 4a	3 (9.70%)	1 (2.90%)	2 (3.50%)	6 (4.80%)	14 (11.30%)
MIH-TNI 4b	0 (0.00%)	2 (5.70%)	2 (3.50%)	4 (3.25%)	
MIH-TNI 4c	1 (3.20%)	1 (2.90%)	2 (3.50%)	4 (3.25%)	
Total count	31 (100%)	35 (100%)	57(100%)	123	123
**Males**					
**MIH-TNI** **Index n (%)**	**10** **years**	**11** **years**	**12** **years**	**Total count**	
MIH-TNI 1	14 (38.90%)	25 (46.30%)	12 (27.30%)	51 (38.00%)	51(38.00%)
MIH-TNI 2a	10 (27.80%)	8 (14.80%)	9 (20.50%)	27 (20.10%)	55 (41%)
MIH-TNI 2b	7 (19.40%)	5 (9.20%)	5 (11.40%)	17 (12.70%)	
MIH-TNI 2c	3 (8.30%)	6 (11.10%)	2 (4.50%)	11 (8.20%)	
MIH-TNI 3	0 (0.00%)	1 (1.90%)	3 (6.80%)	4 (2.90%)	4 (2.90%)
MIH-TNI 4a	0 (0.00%)	1 (1.90%)	2 (4.50%)	3 (2.20%)	24 (17.82%)
MIH-TNI 4b	0 (0.00%)	2 (3.70%)	5 (11.40%)	7 (5.22%)	
MIH-TNI 4c	2 (5.60%)	6 (11.10%)	6 (13.60%)	14 (10.40%)	
Total count	36 (100%)	54 (100%)	44 (100%)	134	134

**Table 5:** Distribution of MIH treatment modalities according to MIH-TNI by age and gender across the 509 affected teeth. Treatment codes are described in the Methodology Section.

Gender	Age	B1	B2	B5	C1	C3	D	E1	E2	F
Males	10 years	37	0	3	1	0	11	4	0	2
	11 years	49	4	11	2	0	29	19	0	1
	12 years	18	2	5	0	1	15	48	2	3
	total number of affected teeth	104 (20.4%)	6 (1.2%)	19 (3.7%)	3 (0.6%)	1 (0.2%)	55 (10.8%)	71 (14%)	2 (0.4%)	6 (1.2%)
Females	10 years	24	4	2	0	0	12	15	0	0
	11 years	32	0	3	0	0	12	22	0	0
	12 years	46	2	18	0	3	18	31	3	2
	total number of affected teeth	102 (20%)	6 (1.2%)	23 (4.5%)	0 (0%)	3 (0.6%)	42 (8.2%)	61 (12%)	3 (0.6%)	2 (0.4%)

## Data Availability

The data that support the findings of this study are available upon request from the corresponding author. The data are not publicly available due to privacy or ethical restrictions.

## Abbreviations

The following abbreviations are used in this manuscript:

DDE	Developmental Defects of Enamel;
EAPD	European Academy of Pediatric Dentistry;
MIH	Molar Incisor Hypomineralization;
MIH-TNI	Molar Incisor Hypomineralization—Treatment Need Index;
PEB	Post-eruptive Enamel Breakdown.
